# Panic disorders: The role of genetics and epigenetics

**DOI:** 10.3934/genet.2018.3.177

**Published:** 2018-07-27

**Authors:** Eun Jeong Kim, Yong-Ku Kim

**Affiliations:** Department of Psychiatry, Korea University Ansan Hospital, Ansan, Republic of Korea

**Keywords:** panic disorder, genetics, epigenetics, polymorphisms, GWAS

## Abstract

Panic disorder is characterized by symptoms with abrupt surges of fear with palpitations, sweating, trembling, heat sensations. Considering its disease burden on each individual and on society, understanding its etiology is important. Though no one specific etiology has been known, like other psychiatric disorders, multiple factors such as genetic, environmental, neurobiological, psychopathological factors have been suggested. In this article, we reviewed currently known etiologies and related study results, regarding especially genetic and epigenetic aspects of the panic disorder. Early studies, including twin studies, family studies, adoption studies suggested highly familial trait of panic disorder. Linkage studies, either, found panic disorder is not a single gene disorder but confirmed existence of multiple related genes. Chromosome and candidate gene studies found few related genes, *NPY, ADORA2A, COMT, IKBKE*. Newer method, genome-wide association studies (GWAS) have been searching for newer genes. No genome-wide significant genes, however, were detected, confirming previously known candidate genes, *NPY5R* on 4q31.3-32, *BDKRB2* on 14q32, instead. Epigenetic modification has also been studied on many different psychiatric disorders. Monoamine oxidase A (MAOA) hypomethylation, taken together with negative life events, showed relation with panic disorder. Glutamate decarbodylases 1 (GAD1) hypomethylation was also specific on panic disorder patients. Relation with noradrenaline transporter (NET) gene *SLC6a2* promoter methylation has also been studied. In conclusion, no specific gene or epigenetic pattern can fully explain etiology of panic disorder. Few genes and epigenetic patterns, however, showed strong association with panic disorder compared to healthy controls. Considering its multivariable background, further studies with larger populations can confirm current results and clarify etiologies of panic disorder.

## 1. Introduction

Panic disorder (PD) is defined with recurrent unexpected panic attacks, indicating abrupt surges of fear with palpitations, sweating, trembling, feeling dizzy, heat sensations and other somatic symptoms. According to Diagnostic and Statistical manuals of mental disorders, fifth edition (DSM-5) panic disorder can accompany symptoms such as nausea or abdominal distress, derealization, fear of losing control, fear of dying [1]. Different from previous version of DSM (Diagnostic and Statistical manuals of mental disorders) diagnosis, current DSM-5 diagnosis criteria separates the panic disorder from the agoraphobia, not as a specifier but as a different independent diagnosis. Another difference, in addition, from previous version of DSM diagnostic criteria is that “with panic attacks” specifier can be applied to all DSM-5 disorders. From these changes, we can assume possible relationships between the diseases.

Panic disorder can deteriorate individual's function with persistent concern or worry about additional panic attacks in daily lives. Regarding studies of past few decades, known 12 month-prevalence rates of PD is ranged from 0.7 to 3.1%, and life-time prevalence from 1.6 to 5.2% [2]–[4].

With its complicated clinical manifestations, patients with panic disorder often visit emergency room or other medical departments, spending their time and money for evaluation and treatment [5]. With its recurrence tendency, they re-visit and their expenditure increases. On this aspect, disability-adjusted life years lost (DALY) were once estimated for panic disorder. DALY represents disease burden including lost due to illness, disability, or early death. In study done at 2005 showed DALY for panic disorder was 383,783, which was smaller than that of depression, dementia but greater than that of Parkinson's disease, epilepsy, which are known to have still big social cost or burden [6]. In study conducted in Italy, panic disorder had impact quality of life of the patients. Study showed lifetime prevalence of PD total 3.6%, with 4.4% in females, compared to 2.5% in males. They also estimated quality of life among patients and compared each other, and patients with comorbid agoraphobia showed statistically worse quality of life [7].

Etiology of panic disorder is still obscure. Genetic, environmental, neurobiological, psychopathological aspects are suggested. Ferris Pitt found hyperosmolar sodium lactate could provoke anxiety neurosis [8], so-called panic attacks in patients with panic disorder but not in healthy controls. After such findings, many substances that can provoke panic disorder have been suggested [9]. These findings could suggest existence of neurobiological etiology of panic disorder, though not confirmed.

Anatomical findings included volume changes of brain, including amygdala, temporal lobe and creatine and phosphocreatine metabolites decrease in medial temporal lobe [10],[11]. Also, decrease in benzodiazepine-receptor density were found near hippocampus or amygdala of patients [12],[13]. In continuum to the findings, gamma-aminobutyric acid (GABA) concentration difference was found on some studies [13]. These findings partially explained neurobiological background of panic disorder.

On psychosocial aspects, key concept to understand etiology of panic disorder is anxiety sensitivity [14]. Anxiety sensitivity is thought to be built from adverse life events, observations, parental modeling of distressed reactions to bodily sensations [15],[16]. Moreover, Panic attacks can enhance anxiety sensitivity. On imaging studies, insular cortex is thought to be related to anxiety sensitivity, thought to reflect bodily awareness [17].

Panic disorder is well-known for sex-difference in its epidemiology. Higher disease prevalence among females, compared to males, is suggested as another evidence of the genetic background of panic disorder. According to studies, male to female ratio of panic disorder ranges diversely. Male to female ratio of 12 months prevalence rate ranged 1.7 to 1.8% and lifetime prevalence rate ranged 1.6 to 2.1% which is higher or similar to that of all other anxiety disorders [4],[18],[19]. It can partly explain with psychosocial factors but biological and genetic factors also contribute this difference. Psychosocial stressors include lower physical strength, chronic stressors, traumatic life events and anxiety sensitivity difference between genders [20]. Neurobiological factors can explain this difference in part, such as serotonergic neurotransmission, beta-adrenergic sensitivity, dopamine pathway differences. Structural/functional neuroimaging also suggests specific brain area or connectivity difference between genders.

These complex etiology of panic disorder makes it difficult to clarify disease entity and furthermore, treatment of the panic disorder. Considering its highly heritable tendency of panic disorder, it would be helpful to understand and treat the patients. In this review, we will discuss, among various etiologies of panic disorder, especially genetic and epigenetic aspects of panic disorder. Clarifying its genetic, epigenetic background and widening understanding of etiology of the disease, thereafter, would help manage panic disorder.

## 2. Genetic aspects of panic disorder

### 2.1. Background and earlier studies

Early studies, including family studies, twin studies, adoption studies and linkage studies, were suggested at the end of 20^th^ century. Family studies evaluated frequency of disorder among probands of patients and compared that of healthy controls. Twin studies evaluated familial distribution, including comparison of monozygotic and dizygotic twins. Adoption studies and linkage studies were performed less in early days but showed important findings. In early family studies, lifetime prevalence was higher among families with patients (7.7 to 17.3%) compared to healthy controls or no-panic groups (0.8 to 8.3%) [21],[22]. Family studies documented increased risk of panic disorder among relatives of affective individuals, 5 to 16% according to studies [23]. Considering first-degree relatives of probands of panic disorder patients, 17-fold increased risk of panic disorder was also discovered [24]. These results suggest considerable heritability of panic disorder. Twin studies also showed concordance between twins regarding emergence of panic disorder. It showed higher prevalence compared to other psychiatric disorders, and also found “symptoms of panic” showed unique genetic influences compared to other anxiety symptoms [25].

Early linkage studies proposed panic disorder is not a single gene disorder, but several genes act together and contribute susceptibility on emergence of disease. It focused the cross sectional result of higher lifetime risk among first-degree relatives. It was assumed that pattern resembled single locus gene transmission pattern [26].

For genetic aspects, few female-specific genetic associations have been reported. Catechol-O-methyltransferase (*COMT*) gene is one of the most frequently reported genes. Specific polymorphism, val158met, showed dominance among female panic patients, supported by gender difference of gene-estrogen interaction. As *COMT* gene codes methylation enzymes metabolizing monoaminergic neurotransmitters, such as dopamine [27], it can also support gender ratio difference. Monoamine oxidase A (*MAOA*) gene [28] has been also found to have different variable number tandom repeat polymorphism. Another difference was found on neuropeptide S receptor gene [29], neuropeptide Y5 receptor gene [30], glutamate decarboxylase 1 and its related genes [31]. These genes showed difference among gender, in consistent with its disease prevalence difference among gender. Considering these gender differences can explain genetic background on etiology of panic disorder.

### 2.2. Chromosomal studies and gene studies

Over 300 candidate genes and related chromosomes are suggested until now [32]. Most studied chromosomal regions include 1q, 4q31-q34, 7p14.3, 9q31, 11p, 13q, 14q, 15q12, 18, 22q. We'll overview some of the study results here. Table 1 also shows findings discussed below.

**Table 1.** Genetic, epigenetic findings and panic disorder

Genetic findings		
Related chromosome	Gene	References
1	*AVPR1B*	[33]
1q31.2, 1q43	*RGS2, RGS7*	[34], [35]
4q31-q34	*NPY (rs11946004)*	[30]
7p14.3	*NPSR*	[42], [29]
9q31	*D9S271*	[43]
11p	*CCKB*	[47]
13q14.2	*HTR2A (rs6313T/C)*	[49], [50]
14q		[51]
15q12	*GABRB3, GABRA5*	[52], [53], [54]
18q		[55]
22	*COMT*	[56]

Epigenetic findings		
Gene and epigenetic change		References

*MAOA* hypomethylation		[75]
Decreased *GAD1* methylation		[78], [79]
*NET* methylation		[80]
*CRHR1* hypomethylation		[81]

One German study assessed 186 patients, 299 healthy controls and examined 71 SNPs in corticotropin releasing hormone (CRH) and arginine vasopressin (AVP) neuropeptide systems, in specific, *CRH, CRHR1, CRHR2, AVP, AVPR1A, AVPR1B*. The results suggested that polymorphisms within *CRHR1*, on chromosome 17, and *AVPR1B*, on chromosome 1, genes had effect on susceptibility for panic disorder [33]. The relation with arginine vasopressin and regulation of anxiety behavior is well in consistent with early animal studies. Another German study assessed *RGS2* and *RGS7* gene, regulator of G-protein signaling genes, located on 1q31.2 and 1q43 each, were both associated with panic disorder, respectively [34],[35]. Another gene on chromosome 1, IKBKE (inhibitor of kappa light polypeptide gene enhancer in B-cells, kinase epsilon) was studied with 210 panic disorder patients, 356 healthy controls [36]. IKBKE stimulates activity of interferon regulatory factor 3, and activates nuclear factor kappa-B signaling pathway [37]. Therefore, IKBKE have known to control the onset of expression of antiviral agents. The study result found SNP rs1539243 was different between panic disorder group and control group. In the same study, depression group also showed similar results, which indicates IKBKE gene to have relations with the comorbid mood and anxiety disorders.

Another risk locus, 4q31-q34 showed another strong associations. This locus is suggested as a region of genes coding for neuropeptide Y (NPY) Y1, Y2 and Y5 receptors. NPY regulates secretion of various hypothalamic neuropeptides, and has vasoconstrictive effects [38]. Its impact on anxiety was first suggested with study with knockout mice. They showed different anxiety behavior, and on study with human, individuals with anxiety showed different levels of NPY plasma levels, especially higher [39]. There exist numerous variables of NPY receptors, most studied ones are NPY, NPY Y1, Y2, Y5. Study found specific variations (Gly-4260Gly) NPY Y5 coding variants, and haplotypes including rs11946004 showed association with panic disorder compared to healthy controls [30]. Rather, comparing to healthy controls, genes coding for NYP Y1, Y2, no significant difference were found. In this manner, evidence for impact of NPY Y5 receptor on emergence of panic disorder can be suggested.

Neuropeptide S is a suggested material for mediating anxiety-related behavior and anxiety disorders. Consisted of 20 amino-acid peptides, neuropeptide S (NPS), is shown to have anxiolytic effect, supported with mice study [40],[41]. NPS receptor (NPSR) knockout mice, therefore, exhibit anxiety-like behavior, together with reduced exploratory behaviors. In human, *NPSR* gene, located on chromosome 7p14.3, has also been studied for its relation with the panic disorder [42]. One German has found specific allele variant, *NPSR* A/T, showed association with panic disorder regardless of existence of agoraphobia [29]. This variant also showed dimensional anxiety traits, autonomic arousal level during behavioral test, all those related with anxiety and related behaviors. Furthermore, with functional activity of brain, T risk allele showed decreased activity in dorsolateral prefrontal, lateral orbitofrontal and anterior cingulate cortex during fearful faces in patients with panic disorder. Regarding these results, chromosome 7p14.3 can be taken into risk loci for panic disorder.

In 2003, there conducted study with 25 extended families, with at least one individual with panic disorder [43]. In linkage analysis studies, LOD score calculated explains how informative the genotype is [43],[44]. If the property lies lower than 1, it's uninformative, and if lies over 1, informative. Linkage analysis resulted in LOD score of 4.18 at D9S271, on chromosome 9q31. When considering families with anxiety disorder, broader than panic disorder, LOD score was still 2.0 suggesting phenotype of anxiety also significantly related to this locus. It can be explained in other aspect, whereas patients diagnosed with panic disorder also have received other anxiety diagnosis. Considering all possible explanations, until now, we can suggest the locus have impact on anxiety related symptoms, not only limited to panic disorder.

On chromosome 11p, there exists cholecystokinin B receptor, 11p15.4. Cholecystokinin is gastrin-like neuropeptide, which modulates interaction between dopamine and other neurotransmitters [45]. When stimulated, for example, it can mimic panic attacks, especially in patient groups. One of CCK receptors is located on chromosome 11p, whereas *CCK-AR* places on 4p15.2-15.1 [46]. One study found difference of length of polymorphic CT repeat allele of *CCK-BR* in patient group compared to control group. In Japanese study, however, showed conflicting results, further studies are required [47].

For chromosome 13, two genes showed possible relation with panic disorder. On 13q region, there exists D-amino acid oxidase activator (DAOA/G30) LG in region 13q33.2 and serotonin receptor 2A (*HTR2A*) in 13q14.2. DAOA/G30 gene is actually known to be related with other psychiatric disorders, such as mood disorders or schizophrenia [48]. Rather, stronger associations were found on *HTR2A* gene polymorphism (rs6313T/C) in specific ethinic samples [49]. Though some studies showed inconsistent results, Korean samples [50] for example, further results are required. Until now, *5-HTR2A* receptors and its panicolytic and anxiolytic effects, when stimulated, is suspected as an underpinning of this results.

There was a large study of anxiety disorder and its linkage study of chromosome 14 [51] in 2008. The study included 1,602 set of twins and siblings, and found heritability at 42%. Especially a LOD score of 3.4 was found on chromosome 14 at 105 cM, with no other regions suggestive of linkage. This finding suggests, when taken previous results all together, the region might harbor QTL (Quantatative Trait Loci) related with general anxiety.

One study found candidate gene at 15q12. This locus is related to gamma-aminobutyric acid type A receptor subunit and patients showed linkage to specific allele of *GABRB3* and *GABRA5*. Relation with *GABR* genes can be explained with biological role of gene and pathogenesis of panic disorder. Regarding well-known disorder Angelman syndrome [52], has *GABRB3* deletion, and role of *GABRA5*, mediating ligand selectivity and affinity [53] and possibly, sedation, these alteration of gene exhibition can alter GABAergic activity. So these genetic variants can explain GABAergic dysregulation and clinical features of panic disorder [54].

Chromosome 18 is one of the candidate chromosome studied from early days in relation with bipolar disorder [55]. In 1998, studies with 28 families that their members were bipolar disorder were conducted. Families were stratified into three groups, with panic disorder, with panic attacks, no-panic attacks. Via linkage analysis, it was found that loci on 18q showed relations with history of panic disorder. It verified that bipolar disorder itself had subtypes divided by genetic factors and also supported that chromosome 18q may show relations with panic disorder susceptibility.

Another chromosome, chromosome 22 has been studied for its relation with anxiety disorders, including panic disorder [56]. Its neurobiological base was existence of *COMT* gene, on chromosome 22, which is an enzyme that catalyzes the extraneuronal methylation of catechol compounds. Higher level of *COMT* was related with “anxiety states” and relation with another psychiatric disorder [57], obsessive-compulsive disorder was also found [58]. Via linkage analysis, elevated LOD scores for specific SNPs were found, and haplotypes were also found. These results supports *COMT* gene or in a nearby region of chromosome 22 can be a susceptibility locus for panic disorder [56]. Neuroimaging study found *COMT* val158met (472G/A) genotype groups had specific activation changes in response to fearful faces on functional MRI [59]. Individuals with at least one 472G risk allele compared to homozygotes 472A allele. Increased activity was found on right amygdala, right fusiform gyrus, and left lateral orbitofrontal cortex, in patients with at least one 472G allele. Especially in response to angry faces, patients with at least one 472G allele showed higher activation in left medial orbitofrontal cortex. The results indicate carrying val risk allele had reduced resilience against anxiety-states in panic disorder, with altered neuronal integration.

When understanding anxiety related models, understanding caffeine and its mechanism of action can help. Caffeine, one of anxiogenic agents that affects on adeonsine A1 and A2A receptors and antagonizes it. As adenosine receptors interact with dopaminergic receptors, these interaction may affect responses to caffeine. One study administered caffeine to healthy controls and found variation of anxiogenic effect on genes for A2A (*ADORA2A*) and DRD2 (*DRD2*). The result showed significant association between caffeine-induced anxiety and specific *ADORA2A* polymorphism (rs5751876, rs2298383, rs4822493) and *DRD2* polymorphism (rs1110976). Result could support that *ADORA2A* and *DRD2* polymorphisms contribute to responses to caffeine.

Genetic and chromosomal studies, however, shows inconsistent results so far. In this section, we reviewed currently studied chromosomes and its relations with the panic disorder. As mentioned above, chromosome 1q and SNP rs1539243 showed positive relation with the panic disorder, also rs11946004 on chromosome 4 showed relations. Chromosome 7p14.3 and chromosome 9q31 also showed positive relations. No specific gene or chromosome, however, showed consistent results throughout studies. It may be due to difference among study designs, ethnic differences. To get precise results, further studies with bigger population are needed.

### 2.3. GWAS studies and future directions

Genome-wide association studies (GWAS) method refers exploring genetic variations of patient and controls, targeting to find, for example, common SNP difference, copy number variants and even rare SNP difference. Different from previous linkage studies, targeting specific, known genes, GWAS can detect unsuspected candidate gene, as a result, help understand genetic background of specific disease. This study design has been widely used in psychiatric field, early studies included diseases such as attention deficit hyperactivity disorder, autism, bipolar disorder, major depressive disorder, schizophrenia [60].

Study for panic disorder has also been conducted. In 2009, Japanese studies with large group of patients were reported. The study included over 300 cases of patients and over 800 controls. The results showed no genome-wide significant SNPs were detected, few loci were detected for having associations with disease [61]. Considering previously known candidate genes, *NPY5R* on 4q31.3–q32 showed relation with disease. *BDKRB2* on 14q32 is another example. *BDKRB2* encodes bradykinin receptor, regulating blood pressure, pain perception [62]. As patients with panic disorder have higher mortality risk related to cardiovascular diseases, clinical importance of this gene can be expected. With weak associations, few more SNPs were discovered, showing multiple SNPs with numerous genes account for development of disease. Few genes, such as *TMEM132D11* and *ACCN1*, however, showed the opposite results compared to previous studies [63],[64]. *TMEM132D11*, for example, were presented as a candidate gene for panic disorder, but the study showed no difference between the patient group and the control group. *ACCN1*, either, showed no relation in this gene-wide levels of significance. Factors such as difference in ethnicity needs be taken into account.

Japanese group, further published another meta-analysis paper done with two GWAS data sets, including 718 cases and 1717 controls [65]. Study assessed all the candidate gene analyses using both GWAS and meta-analysis results. Genome-wide significant SNPs were not detected, however, suggestive associations were found on *BDKRB2*, which was also previously mentioned. The strongest evidence of association was found at rs12501691 in *NPY5R* on 4q31.3-q32. Associations reported significant only on female samples, consistent on previous results. Neuropeptide Y (NPY) had modulating effect on stress exposure on susceptibility to anxiety disorders in both animal models and human studies. This difference in *NPY* gene expression can therefore contribute to anxiety disorders, including panic disorder. *TMEM132D* and *ACCN1*, however, didn't show association with panic disorder. Though small evidences for associated gene were found, no genome-wide significant SNPs were found.

Studies until now have relatively small effect size for example, for GWAS, or inconsistent results, further studies are required. Future studies should focus more on studies with larger sample to confirm the previous results. Thinking of shortcomings of GWAS design itself, we should take it into account that possible genes might have been not detected. For SNPs with less effects or less allele frequencies, studies with small sample sizes cannot detect such findings [65]. When interpreting such study results, precise consideration is required.

Also, studies should focus more on specific symptoms of each patients, rather than disease itself. Furthermore, specific gender-specific or family-specific disease architectures could be found with genotype-phenotype specific studies [66]. Development of next generation DNA sequencing technologies would also help newer research techniques. Combining genetic factors with environmental factors, so-called Gene x Environment interaction is another way to study etiologies of the disease. Results from all these studies can help clinicians to apply individualized care, and therefore help threatened patients.

## 3. Epigenetic aspects of panic disorder

Epigenetics has recently emerged as a potential mechanism by which adverse external stimuli such as early life trauma, can alter gene expression in later lives [67]. It refers to modulatory mechanisms that operate above the nucleotide bases that comprise a gene's DNA sequence. Epigenetic operations work on sequences that are not encoded on gene's DNA sequence [68]. It is of importance for understanding modifiable impact of environment on genetic factors though it is not encoded innately. Well-known processes usually include DNA methylation, histone acetylation, microRNA dysregulation [69].

Many studies have been already done for psychiatric disorders about epigenetic process on disease course. For example, for depression, early life stress influenced adversely on epigenetic modification of gene expression. Antidepressant treatments could reverse epigenetic process of disease. These alterations could impact various steps of genetic expressions, from gene expression to protein synthesis. Most studied epigenetic modification step is DNA methylation. Common site of methylation is among gene promotor regions, cytosine-phosphate-guanine dinucleotide (CpG) site [70]. The site is thought to regulate, usually suppress gene expression by blocking binding of enzymes that work for RNA synthesis. Environmental factors could modify this step and alter gene expression. Another modifiable step includes histone acetylation, methylation, microRNAs, and its biogenesis. Example of environmental factors included early life stressors, including pre- and post-natal stressors. As epigenetic modification can be done with antidepressant treatments [71], its clinical significance stands for exploration of newer treatment options, as already tried on other medical fields [72]. Epigenetic aspect on etiology of panic disorder is not well studied compared to other factors such as genetic aspects or environmental factors. Overview of current study results will be discussed here.

MAOA contributes to degradation of monoamines, such as epinephrine, norepinephrine, serotonin, with oxidative deamination process [73]. Especially *MAOA* gene has been identified as a potential risk gene for panic disorder. Considering treatment response difference according to different gender and allele difference in specific chromosome loci [74], its role on panic disorder have been suggested. According to studies to date, not specific allele itself, but methylation patterns in the regulatory regions of the gene were highlighted. On *MAOA* promoter/exon 1/intron 1 CpG sites, according to study with 65 patients, 65 controls, all studied male had non-/marginally methylated form of methylation, while females all showed variable levels of methylation [75]. Furthermore, methylation rate also showed relation with panic disorder emergence, hypomethylation on female showed relation with disease. Such pattern of *MAOA* gene hypomethylation in panic disorder is in concordance with that in anxiety disorder or depressive disorder. Considering factors such as negative life events, predictability on susceptibility of disease becomes more precise.

Like a depression, treatment could reverse epigenetic risk pattern of panic disorder [76]. There was a trial of studying *MAO* gene, hypothesizing temporal plasticity of epigenetic processes that *MAOA* methylation changes during psychotherapy. The study tried cognitive behavioral therapy (CBT) especially exposure-based one for panic disorder patients. CBT was conducted for 6 week session. At baseline, *MAOA* hypomethylation was dominant on panic disorder patient group compared to healthy control group. Along with CBT response, *MAOA* methylation increased, up to level of healthy controls. Clinically, increase of *MAOA* methylation showed correlation with, especially, agoraphobic symptom reduction. This is in consistent with results shown in depression, treatment influences modification or reverse of epigenetic processes. These epigenetic findings, in addition, suggest similar characteristics of different psychiatric disorders with the panic disorder, suggesting possible epigenetic effect on evolution of disease.

Glutamate decarboxylases (GAD67/65; GAD1/GAD2) are enzymes influence on gamma-aminobutyric acid (GABA) synthesis, thereby dysfunction on *GAD1/GAD2*, causes dysfunction on GABA system. As GABAergic system mainly constructs neurobiological background of emergence of anxiety [77], epigenetic processes related to *GAD1/GAD2* genes is clinically of importance. Several single nucleotide polymorphisms in *GAD1* gene are associated with variety of anxiety disorders, including generalized anxiety disorder, panic disorder, agoraphobia and depression. Specific SNPs and haplotypes (SNPs, rs769407, rs3791851, rs769395) showed phobias and *GAD1* rs3749034 showed association with panic disorder in female population [78],[79]. Recent study of epigenetic processes, the study included 65 patients with panic disorder and matched healthy controls, and assessed DNA methylation status at 38 *GAD1* promoter/intron 2 and 10 *GAD2* promotor CpG sites. *GAD1* methylation was low on panic patients group compared to healthy controls. Especially female patients, negative life events showed more correlation with decreased methylation. For *GAD2* gene, no specific difference was found. These results suggest possible epigenetic process on panic disorder, mediating influence of negative life events.

Variation in the noradrenaline transporter (NET) gene *SLC6a2*, especially methylation was once explored [80]. *SLC6a2* promotor methylation was assessed in both panic disorder group and control groups, both before treatment and after treatment. Though study found that methylation rate was not different between groups, methylation levels at some CpG could showed correlation with few physiological measures, such as blood pressure, heart rate, age, body mass index (BMI) and arterial concentrations of noradrenaline and its metabolite DHPG. As considering its role in NET expression, we assume it can somehow affect mood, anxiety or related symptoms.

More recent studies suggest the role of corticotropin releasing hormone receptor 1 (CRHR1) hypomethylation in panic disorder [81]. CRHR1 is involved in hypothalamic-pituitary-adrenal axis and related stress response. The study compared healthy controls and the panic disorder patients and result showed relation with hypomethylation of CRHR1, in other words, increased expression of CRHR1 in the panic disorder patients.

## 4. Conclusions

Understanding complex etiology of the panic disorder is difficult and so far, no clear genetic or epigenetic etiologies have been discovered. One of the most studied and supported gene with multiple results is *COMT* Val158Met polymorphism, especially in female panic disorder patients. Many genes, such as *ADORA2A, NPY, DRD2, GABRB3*, has shown relation with panic disorder in few studies, with supporting evidence of each gene having relation with panic disorder or, in part, anxiety related function. Via GWAS method, no genome-wide significant SNPs were detected, but we could confirm few previously known to be related genes. Fewer epigenetic studies have been conducted. *MAOA* methylation rate has shown significance in patient group, affected by negative life events and genders. Treatment could reverse methylation pattern of patient group, and other epigenetic processes, such as low *GAD1* methylation was found in patient group, also on those with female gender and previous negative life events. Future studies should include larger population to confirm current inconsistent study results, and it will help us to understand complexed etiology of panic disorder.
